# Inhibition of preS1-hepatocyte interaction by an array of recombinant human antibodies from naturally recovered individuals

**DOI:** 10.1038/srep21240

**Published:** 2016-02-18

**Authors:** Anurag Sankhyan, Chandresh Sharma, Durgashree Dutta, Tarang Sharma, Kunzang Chosdol, Takaji Wakita, Koichi Watashi, Amit Awasthi, Subrat K. Acharya, Navin Khanna, Ashutosh Tiwari, Subrata Sinha

**Affiliations:** 1 Department of Biochemistry, All India Institute of Medical Sciences, New Delhi, India; 2 Center for Bio-design, and Diagnostics Translational Health Science and Technology Institute, Faridabad, Haryana, India; 3 Department of Virology II, National Institute of Infectious Diseases, Shinjuku, Tokyo, Japan; 4 Center for Human Microbial Ecology, Translational Health Science and Technology Institute, Faridabad, Haryana, India; 5 Department of Gastroenterology, All India Institute of Medical Sciences, New Delhi, India; 6 Recombinant Gene Products Lab, International Center for Genetic Engineering and Biotechnology, New Delhi, India; 7 National Brain Research Center, Manesar, Gurgaon, Haryana, India; 8 Experimental Medicine and Biotechnology Department, Postgraduate Institute of Medical Education and Research, Chandigarh, India

## Abstract

Neutralizing monoclonal antibodies are being found to be increasingly useful in viral infections. In hepatitis B infection, antibodies are proven to be useful for passive prophylaxis. The preS1 region (21–47a.a.) of HBV contains the viral hepatocyte-binding domain crucial for its attachment and infection of hepatocytes. Antibodies against this region are neutralizing and are best suited for immune-based neutralization of HBV, especially in view of their not recognizing decoy particles. Anti-preS1 (21–47a.a.) antibodies are present in serum of spontaneously recovered individuals. We generated a phage-displayed scFv library using circulating lymphocytes from these individuals and selected four preS1-peptide specific scFvs with markedly distinct sequences from this library. All the antibodies recognized the blood-derived and recombinant preS1 containing antigens. Each scFv showed a discrete binding signature, interacting with different amino acids within the preS1-peptide region. Ability to prevent binding of the preS1 protein (N-terminus 60a.a.) to HepG2 cells stably expressing hNTCP (HepG2-hNTCP-C4 cells), the HBV receptor on human hepatocytes was taken as a surrogate marker for neutralizing capacity. These antibodies inhibited preS1-hepatocyte interaction individually and even better in combination. Such a combination of potentially neutralizing recombinant antibodies with defined specificities could be used for preventing/managing HBV infections, including those by possible escape mutants.

Around 350 million chronically infected individuals constitute the global disease burden of Hepatitis B and its related complications such as liver cirrhosis, liver failure and hepatocellular carcinoma (HCC). The high endemicity zones include Asia, Africa, southern Europe and Latin America. Global vaccination programs over the past two decades with the viral small surface protein (SHBs) containing vaccine have considerably decreased the overall incidence of HBV infection^1^,^2^. Also, more than 90% of adult-acquired HBV infections are spontaneously cleared by the qualitatively and quantitatively strong immune responses^3^. However, low HBV vaccine coverage of viral mutants, vertical transfer from infected mother, accidental exposures through needle-stick injuries, liver transplantation and immune-compromised individuals accompanied with an increase in ‘viral escape mutants’ are responsible for the high global prevalence of this disease^2^,^4^.

The current post-exposure prophylactic measures advocate the use of plasma derived hepatitis B immune globulin (HBIG) in combination with HBV vaccine in the cases of accidental/perinatal HBV exposure^5^,^6^. But the associated issues of risk of blood-borne cross contamination, cross reactivity, low specific activity, limited availability and cost-effectiveness are some of the growing concerns over its long-term use^7^,^8^. The presence of disease concomitantly with an apparently neutralizing antibody response has been an argument against the use of anti-HBs antibodies in therapy. However neutralizing efficacy of anti-HBV antibodies has been demonstrated in non-human primates^9^,^10^,^11^,^12^.

The small Hepatitis B surface (SHBs) protein is the major component of the viral envelope which constitutes the immunodominant epitope (‘a’ determinant) on viral envelope and elicits maximal neutralizing humoral response. However the virus evades this response by the secretion of non-infectious spherical or filamentous sub-viral particles in 1000–10000 fold excess over the infectious virions. These decoy particles mimic the virions and soak up the virus specific immune response, thereby, reducing its efficacy^3^,^13^. The spherical decoy particles are solely composed of SHB protein and its large excess over the virus makes anti-SHB antibodies less effective and are, therefore not an ideal choice for passive immunization. The other two surface proteins, middle Hepatitis B surface (MHBs) and large Hepatitis B surface (LHBs) proteins, have additional PreS2 (55a.a.) and PreS1 (119/108a.a.) + PreS2 regions respectively at the N-terminus of the SHBs. The other decoy particles, secreted in small proportion-filamentous particles, include MHBs and LHBs proteins in addition to SHBs (L:M:S = 1:1:4)^14^. The preS1 protein which contains the viral putative hepatocyte binding domain between 21–47 amino acids is crucial for viral attachment and entry into the hepatocytes^15^,^16^,^17^,^18^,^19^,^20^. The sodium taurocholate cotransporting polypeptide (NTCP) membrane transporter expressed by the hepatocytes has recently been reported as the functional HBV receptor. NTCP specifically interacts with the putative hepatocyte binding domain in the preS1 region of LHBs of HBV. NTCP can therefore serve as a target for developing therapeutic anti-HBV agents^21^,^22^,^23^. However the classical target, the viral hepatocyte binding domain has shown that anti-preS1(21–47 a.a.) agents such as antibodies are quite effective in neutralizing the virus by blocking its attachment, endocytosis and possibly membrane penetration into the hepatocyte^9^,^10^,^11^,^24^. A number of highly specific murine and humanized preS1-specific monoclonal antibodies that effectively neutralize HBV infection in non-human primates have been generated^9^,^10^. The preS1 component has, therefore been incorporated into the third generation hepatitis B vaccines to achieve a stronger neutralizing response against the virus^25^.

Monoclonal antibodies (murine or chimeric), revolutionized the antibody based therapeutics, but the ability of these antibodies to generate human anti-murine antibody (HAMA) response makes them less preferred than humanized or human antibodies^26^. Recombinant human antibodies are promising alternative to these antibodies. These antibodies offer a major flexible advantage of gene manipulation and antibody engineering to produce antibody variants with better functional characteristics such as lower immunogenicity, higher affinity, specificity and enhanced stability^27^,^28^. Phage-displayed recombinant antibody libraries from human source (naïve, infected or recovered individuals), containing >10^8^–10^10^ antibody sequences can be generated and high affinity monoclonal antibodies can be screened from them against specific target antigens^29^,^30^. Immune libraries from spontaneously recovered individuals present a greater probability of obtaining high affinity and specific neutralizing antibodies.

High viral mutation rates along with subtle variations in viral genotype sequences may render the monoclonal antibody therapy less efficient. We therefore, aimed to generate an array of antibodies with overlapping binding specificities, broadly covering a single large epitopic region. In this article, we report the generation of potentially neutralizing preS1-specific recombinant human monoclonal scFvs from the peripheral blood lymphocytes of individuals spontaneously recovered from hepatitis B. Four scFvs with unique variable region sequences and binding sites were screened and each one showed a distinct molecular interaction pattern with the peptide. All four scFvs individually and even when used in combination were able to block binding of the preS1-peptide to hNTCP, the recently discovered hepatocyte receptor for HBV, in HepG2-hNTCP C4 cells. We suggest that the combined usage of these recombinant human antibodies may achieve broad spectrum neutralization and in addition provide better post-exposure prophylactic interventions for HBV infection.

## Results

### Anti-preS1 antibodies are present in the serum of spontaneously recovered individuals

**Figure 1:**
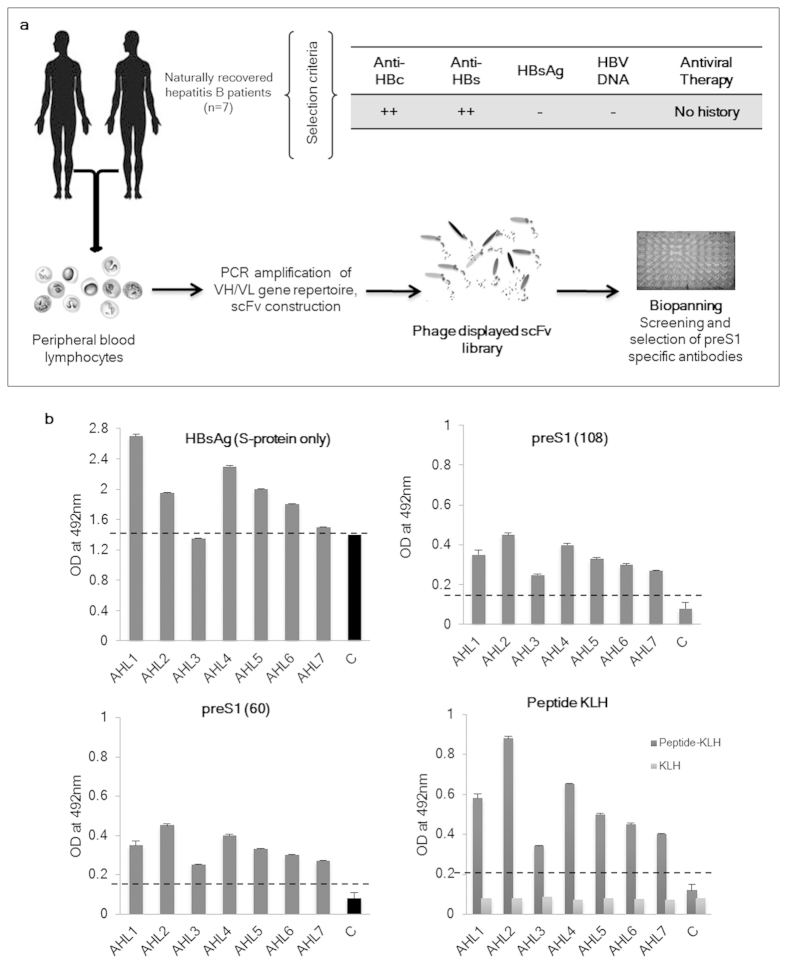
Generation of preS1 specific antibodies from naturally recovered patients. (**A**) Schematic overview of the generation of recombinant phage displayed monoclonal antibodies from naturally recovered hepatitis B patients. (**B**) The results of human serum ELISA with immobilized HBsAg VLPs, recombinant preS1(60), recombinant preS1(108) and the preS1-peptide-KLH conjugate. KLH was used as a control antigen while serum from a vaccinated individual (with the HBsAg S-protein only containing vaccine) was used as a healthy control. The cutoff value is shown in each graph by dotted line (mean + 2 S.D.) of the control samples.

We reasoned that individuals who have recovered naturally from hepatitis B infection would have clonally selected circulating memory B cells/plasma cells secreting highly specific neutralizing antibody against HBV. A flowchart that outlines the strategy used for the generation of preS1 specific monoclonal antibodies from a cohort of such patients is shown in Fig. 1A. Seven individuals with chance detection of anti-HBc antibodies during blood donation screening were further examined for hepatitis B infection markers. All the individuals tested positive for anti-HBc and anti-HBs antibodies but were HBsAg and HBV DNA negative, and since none of them had any history of any therapy for the infection, they were recruited for the study. The results were consistent with the available literature and suggested a previous exposure to hepatitis B virus followed by natural recovery from the infection. Further analysis of the sera from these individuals for HBV specific antibodies was done using an indirect ELISA with immobilized HBsAg (containing S-protein only), purified recombinant preS1(60) and preS1(108) antigens and the preS1-peptide-KLH conjugate. This deciphering of the anti-HBs response showed that a strong anti-S protein response, which generally forms the memory response, was present in all the individuals. In addition, antibodies against preS1 region were also present which reacted with the recombinant full length preS1 protein (108 a.a.), truncated preS1 protein containing N-terminus 60 amino acids as well as the 28-mer preS1-peptide (21–47 a.a.). Anti-HBs as well as anti-preS1 antibodies were detected in the sera of all the individuals. Control serum from a healthy vaccinated individual (with only S-protein containing vaccine) did not show any preS1 response but had elevated levels of anti-HBs antibodies (Fig. 1B).

### Phage displayed scFv library from naturally recovered individuals yields human anti-preS1 antibodies

**Figure 2:**
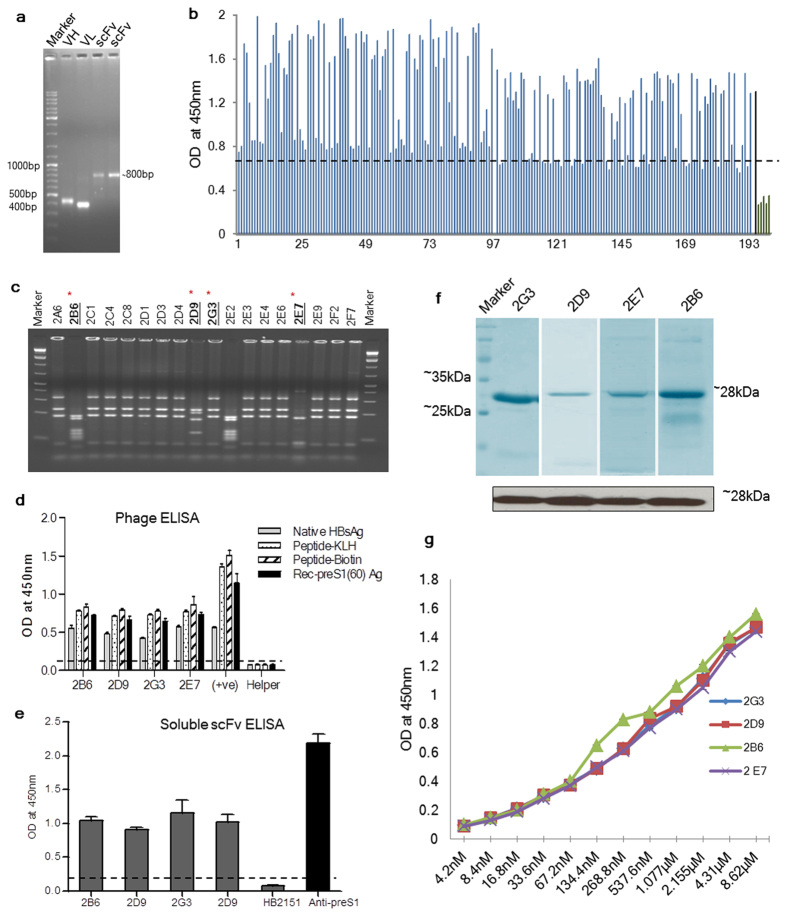
scFv library construction and biopanning. (**A**) Representative figure showing the pooled VH and VL repertoires amplified from the RNA isolated from the blood of individuals naturally recovered from hepatitis B and the final constructed scFv which was cloned into pAK100 phagemid vector for scFv library generation. (**B**) Screening ELISA of 192 different phage clones (picked after third round of biopanning) against biotinylated preS1-peptide. The bound clones were detected using an anti-M13 HRP conjugated antibody. Anti-preS1 polyclonal antibody was used as a positive control while VCSM13 helper phage was used as a negative control (Shown in black). Each bar represents an individual clone. Clones with mean values 2.1 fold greater than mean of negative controls were taken as positive binders. (**C**) Determination of sequence variability amongst the selected round 3 monoclonals using BstNI DNA fingerprinting of amplified scFvs. Four different fingerprinting patterns obtained from the screened 18 clones are underlined. (**D**) Phage ELISA of four antibodies selected after screening and BstNI fingerprinting, to different preS1 containing antigens: Native HBsAg; Biotinylated preS1-peptide; preS1-peptide-KLH conjugate and recombinant preS1(60) coated on a Nunc Maxisorb plate. The Rabbit anti-preS1 polyclonal antibody was used as a positive control while helper phage as a negative control. (**E**) ELISA of the soluble scFvs (using periplasmic extract) to preS1-peptide; HB2151 periplasm is used a negative control; anti-preS1-peptide (21–47 a.a.) rabbit polyclonal antibody was used as a positive control. For Fig. 2D,E, the cut-off values have been taken as mean of the control samples +2(S.D.). (**F**) SDS-PAGE (upper panel)and western blotting (lower panel) of the four scFvs purified using Ni-NTA affinity chromatography.(**G**) ELISA of the four scFvs with the preS1-peptide. Two fold dilutions of each scFv were incubated with the biotinylated preS1-peptide coated on a streptavidin coated plate and detected using anti-his rabbit antibody.

The presence of anti-preS1 antibodies in the sera of individuals naturally recovered from hepatitis B confirmed that the probability of obtaining preS1 specific neutralizing antibodies was high from these individuals. Amplification of the entire repertoire of heavy and light chains using RNA extracted from the PBLs of these individuals was carried out using a set of 31 consensus primers. The pooled V_H_ and V_L_ repertoire products ( ~ 400 bp each) were linked by a flexible (Gly_3_Ser)_4_ linker to construct an scFv (visualized as a band of ~800 bp) (Fig. 2A). The scFv was further cloned into a phagemid vector (pAK100) to construct an immune phage displayed scFv library. Using 1 μg ligation reaction and transformation into TG1 *E. coli* cells, we were able to obtain a compact scFv library consisting of ~1 × 10^5^ different scFv molecules, while no clones were observed in no-insert ligation. Random screening of unselected library clones showed 100% insert frequency and also significant sequence variability on BstNI digestion and sequencing (data not shown). Biopanning of this library against the preS1-peptide yielded more than 300 monoclonals which were screened against the biotinylated preS1-peptide by an indirect phage ELISA and high-binding specific scFv clones were identified (Fig. 2B). The best 18 high affinity clones were further analyzed for sequence variability using BstNI digestion. Among these eighteen scFvs, only 4 different fingerprinting patterns were observed; 14 out of 18 clones had similar fingerprinting patterns (similar to 2G3), while two other clones, 2E2 and 2B6, had identical fingerprints. Clones 2D9 and 2E7 had unique fingerprinting patterns (Fig. 2C). Phages from these four clones 2B6, 2D9, 2E7 and 2G3 were rescued, amplified, PEG precipitated and their binding analysis was performed with different preS1-containing antigens. Despite the complexity and source of the antigens used (plasma purified HBsAg, preS1-peptide-KLH conjugate and purified recombinant preS1(60) and preS1(108) antigens), all the antibodies bound specifically to the antigens that incorporated the preS1-peptide. The specific binding was confirmed by the lack of binding of these scFvs to the control antigens, KLH and Thioredoxin (Data not shown). The binding affinities of all the four phage-scFv (10^12^ phages/well) were similar to different antigens in an indirect phage ELISA (Fig. 2D).

Properly folded, functional and soluble (phage-free) scFvs were obtained by expression of scFv-pAK400 constructs in the periplasmic space of HB2151 *E. coli* host. The periplasmic extract from all the clones showed specific high affinity binding to the biotinylated preS1-peptide in an indirect ELISA. Rabbit polyclonal antibody specific to the anti-preS1-peptide (21–47 a.a.) and HB2151 periplasmic extract were used as positive and negative controls respectively (Fig. 2E). Single step purification of the antibodies from the periplasmic fraction was carried out by Ni-NTA affinity chromatography but the overall yield of the purified scFvs was low; for purified 2B6, 2E7 and 2D9 scFv the yield was  <1 mg/L culture, while for purified 2G3 the yield was ~5 mg/L culture (Fig. 2F). For large scale production in a prokaryotic system, further engineering and manipulation of expression conditions of individual antibodies is required. The identity of the purified scFvs was confirmed by immunoblotting and detection using anti-6XHis antibody (Fig. 2F). Two-fold serial dilution of the purified antibodies was done and ELISA was carried out with the biotinylated preS1-peptide where all four scFvs showed similar binding characteristics despite their sequence variability (Fig. 2G).

### Antibodies show differential germline usage and sequences

The antibody diversity at germline level is limited to a few germline sequences but during the affinity maturation process, mutations are accumulated in these sequences via rearrangement of V, D, and J segments, differential VH/VL pairing, addition or deletion at the junctional site and somatic hypermutation resulting in antigen-specific antibodies. The sequences of the four selected antibodies varied considerably and also, the CDRs accumulated more mutations in their sequences. Analysis of heavy and light chain germline usage among the four antibodies using V-Quest at IMGT^31^,^32^ (http://www.imgt.org/IMGT_vquest/share/textes/) showed that all four scFvs were highly mutated in the variable region with respect to their germline counterparts.

A high ratio of replacement (R) to silent (S) mutations in an antibody sequence is indicative of the antibody undergoing somatic hypermutation process. The four scFvs showed differential heavy chain germline gene usage with IGHV6 being used in 2E7 and 2G3, while 2B6 and 2D9 scFvs used IGHV3 and IGHV4 respectively. Diversity of the VL part of the paratope was however smaller with only IGKV1 being represented in all but for one occurrence of IGKV3 (in 2E7 mAb). The 2G3 V_H_ was found to be of highest homology with germline V_H_ gene IGHV6-1^*^01 F with 92.2% identity in terms of nucleotides. A total of 29 mutations were observed with 18 replacement (R) and 11 silent (S) mutations in a ratio of 9/3 and 9/8 (R/S) for CDRs and FRs, respectively. The 2G3 V_L_ shows 87.46% identity (at nucleotide level) with the germline light chain gene IGKV1-12*01. Out of the total 38 nucleotides changed from germline genes, 27 cause replacement mutations in the V-region (nine of which are clustered in CDRs). The R/S mutation ratio for light chain are 10/4 and 17/7 for CDRs and FRs respectively. 2B6 V_H_ was a result of recombination between Homsap IGHV3-11^*^01 V-gene (89.9% homology), Homsap IGHJ1^*^01 J-gene (84.78% homology) and Homsap IGHD2-2^*^01 D genes. The productive 2B3VL resulted from a recombination between Homsap IGKV1-12^*^01 (91.4% homology) and Homsap IGKJ3^*^01 (89.4% homology) from the V and J-regions respectively. 2D9 V_H_ and V_L_ have high homology of approximately 97% each (at nucleotide level) to their germline counterparts. Heavy chain gene may have originated from the recombination of IGHV4-b^*^02 F, Homsap IGHJ5^*^02 F and Homsap IGHD7-27^*^01 F genes while the recombining V and J genes in 2D9 V_L_ are Homsap IGKV1-27^*^01 and Homsap IGKJ1^*^01 F respectively. The homology search identified Homsap IGHV6-1^*^01 F as the V-gene allele with a homology of 95.2% to 2E7V_H_ while the recombining J and D genes were identified as Homsap IGHJ6^*^02 F and Homsap IGHD1–14^*^01 respectively, showing an identity of 80%. The light chain was a result of recombination between Homsap IGKV3–20^*^01 F (93.26% homology) and Homsap IGKJ2^*^01 F (84.62% homology) genes from V and J regions respectively.

**Figure 3:**
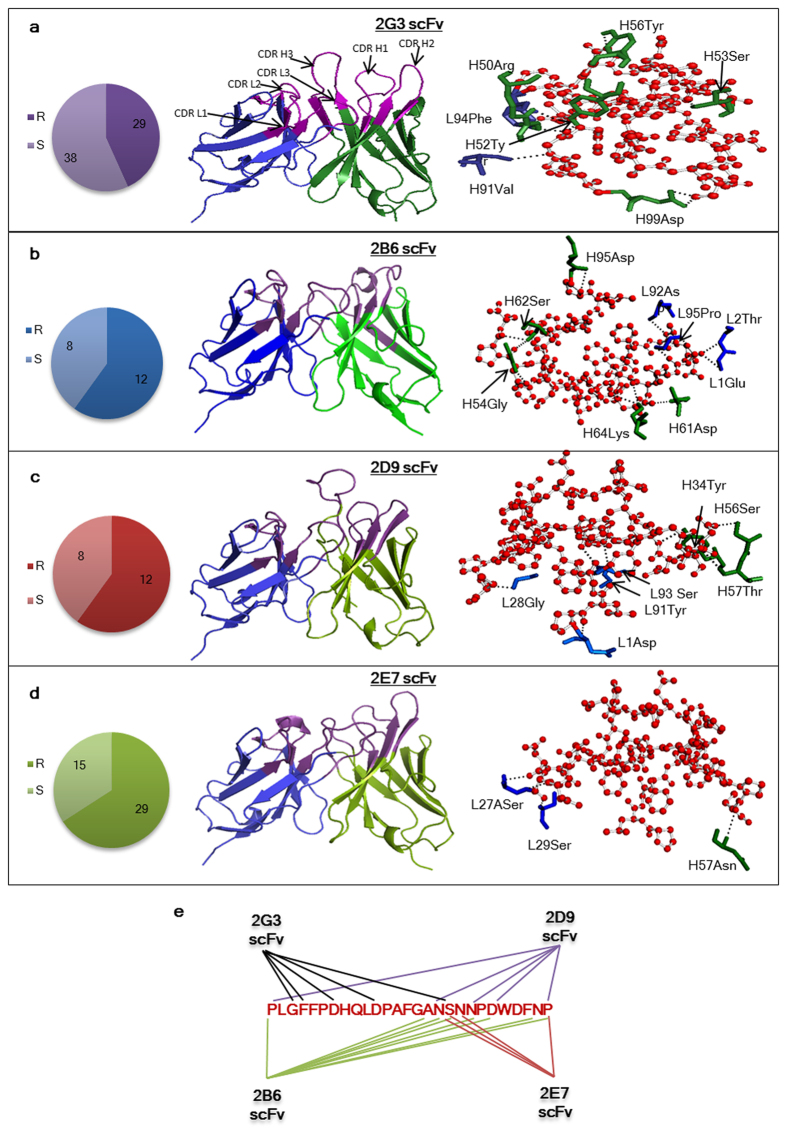
PreS1-peptide-scFv interactions. (**A**–**D**) Panel 1: The replacement (R) and substitution (S) mutations in each clone at the nucleotide level in the variable region of the antibodies. Panel 2: The molecular models of four scFvs. CDRs loops forming antigen binding sites of different topology in each scFv are highlighted. Panel 3: Ball and stick representation of the preS1-peptide-scFv interaction. The interacting residues of the heavy chain are shown in green while the light chain residues are in blue while the peptide is represented as red. The dotted lines show the interactions of the peptide and scFv residues in each case. (**E**) A summarized model of broad coverage of entire preS1-peptide region (21–47 a.a.) by recognition of overlapping epitopes by the four scFvs.

The R/S mutations in the heavy chain of 2B6, 2D9 and 2E7 are 23/8, 7/2 and 12/8 respectively, while that for the light chain are 20/9, 5/6 and 17/7 respectively. The number of replacement and silent mutations and the germline genes of the heavy and light chains of all the four scFvs have been summarized in Fig. 3 and Table 1.

### Molecular modeling of the anti-preS1 antibodies and preS1-peptide

**Figure 4:**
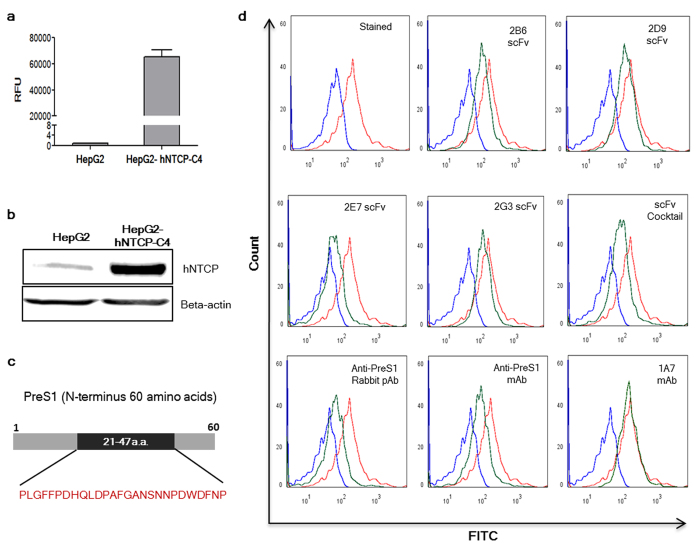
(**A**) NTCP expression levels in the HepG2 cells and the HepG2-hNTCP-C4 cells using real time PCR. (**B**) Western blot showing the relative NTCP protein expression levels in the two cell lines; Bottom panel: Beta-actin for both the cell lysates. (**C**) Schematic showing PreS1 (N-terminus 60 amino acids) and the location of the peptide between 21–47 amino acids. (**D**) Representative flow cytometry experiment showing binding of the preS1-peptide to the HepG2-hNTCP-C4 cells and inhibition of the preS1-peptide-hNTCP interaction by the four antibodies individually and as a cocktail. The rabbit polyclonal antibody to preS1peptide (21–47 a.a.), anti-preS1 RpAb, and a commercial anti-preS1 mAb were taken as positive controls while an unrelated mAb, 1A7, was taken as a negative control. Student’s t-test was used for the statistical analysis and p-values < 0.05 were considered significant.

In the absence of solved crystal structures, near-native structures of antibodies and antigens can be deciphered by molecular modeling based on homology with known structures. Web-based molecular modeling server, web antibody modeling server, WAM^33^ (http://antibody.bath.ac.uk/) was used for the generation of molecular models for 2D9 and 2G3. WAM model could not be predicted for 2E7 and 2B6 since the vernier residues in these scFvs were not conserved. Prediction of Immunoglobulin Structure, PIGS^34^ (http://arianna.bio.uniroma1.it/pigs) server was used for building the scFv model using default parameters for these scFvs. The CDRs of all scFvs attained the characteristic loop structures, forming distinct antigen binding pockets with unique surface topology. Figure 4 shows the cartoon representation of the four antibodies with highlighted CDRs. The structural model for preS1-peptide (21–47 a.a.) was predicted by the web based molecular modeling server, ***i-Tasser***^35^ (http://zhanglab.ccmb.med.umich.edu/I-TASSER/). Model1 was the best predicted structural model with a confidence score (C-score) of 1.94; TM-score = 0.48 ± 0.15; RMSD 5.3 ± 3.4 Å by the server. The molecular model analyzed by Pymol showed that the peptide attained a loop conformation in solution and any other secondary structures were absent.

### Differential interactions of preS1-peptide residues with selected HuMAbs

To study the detailed molecular interactions involved in peptide recognition by the four antibodies, docking experiments were carried out using the modeled peptide and the WAM/PIGS model of the scFv on the antigen-antibody interface of the web based docking server ‘*Patchdock’*^36^ (http://bioinfo3d.cs.tau.ac.il/PatchDock/). Figure 4 gives a ball and stick representation of the interacting residues of the peptide and the four scFvs. The predicted interacting residues have been listed in Table 2. Seven residues of 2G3 from three different CDRs and one FW region contributed to the overall interaction with five residues of the peptide. CDRH2, CDRH3 and CDRL3 loops formed the antigen binding pocket with one vernier residue from the FW4 contributing to the lone FW interaction with the peptide. The residues from CDRH2 loop are predicted to provide the maximal contribution to peptide binding with 3 CDRH2 residues forming polar contacts with the residues of preS1-peptide.

The putative epitopic footprint of the 2B6 antibody spans from residue 16–27 on the preS1-peptide which is distinct from 2G3 epitopic footprint. The CDRH2 and CDRL3 provide the major contributions towards antigen binding with 2 and 3 residues from FRL1 and FRH3 respectively also putatively interacting with the preS1 peptide.

The predicted epitopic footprint of the 2D9 mAb is similar to 2B6 but the peptide interacting residues are fewer. The Asn17 residue from the peptide interacts with two CDRH2 residues while the CDRH1 residue H34Tyr interacts with the Asn20 on peptide. Two light chain residues from CDRL3 form an H-bond each with the Asp22 residue while the CDRL1 residue L28Gly interacts with Pro27 residue on the peptide.

The docking studies further predicted that 2E7 scFv forms only 3 bonds with the peptide spanning the residues 18–27. The Pro27 residue which is common interacting partner of the 2B6 and 2D9 also interacts with the 2E7 residue H57Asn from CDRH2 while two Ser residues from CDRL1, interact with Ser18 and Asn19 residues on the peptide respectively. The number of interactions formed by different antibodies is different but the binding strength of all the antibodies is similar.

### *In vitro* ‘surrogate neutralization assay’ shows inhibition of peptide-hNTCP interaction by scFv antibodies

**Figure 5:**
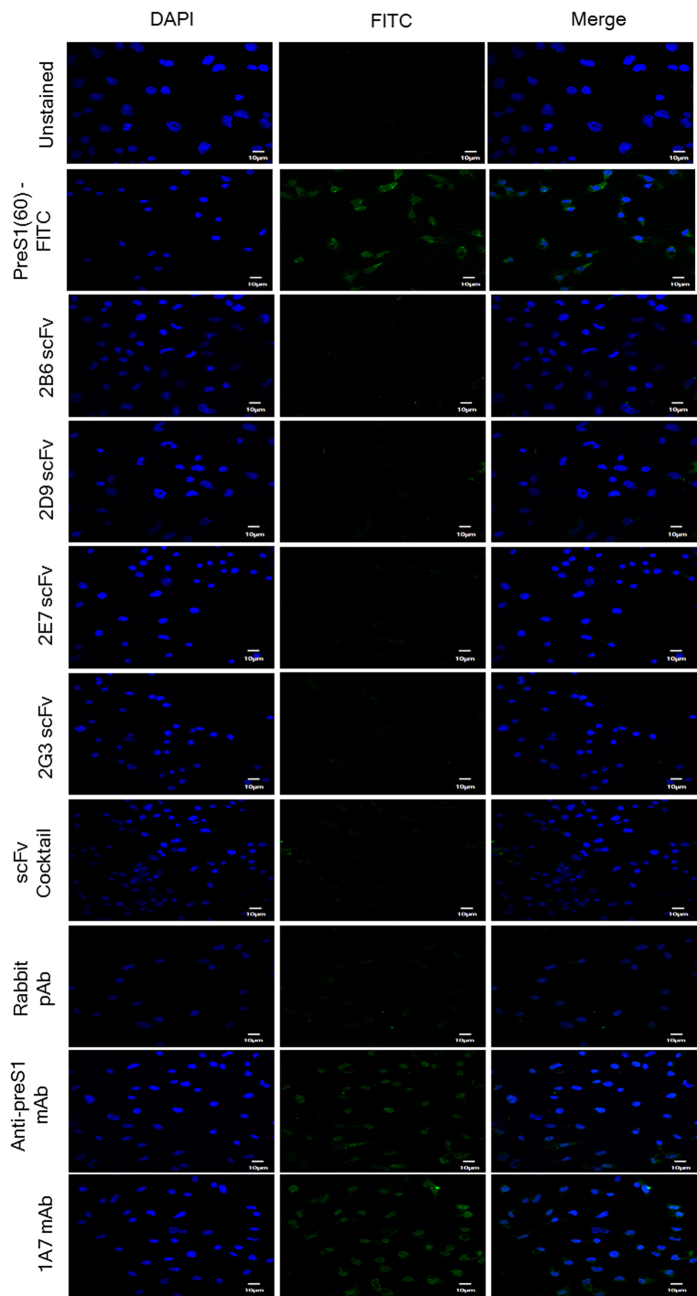
(**A**) Fluorescence microscopy images (using confocal microscope) showing inhibition of peptide-HepG2-hNTCP-C4 cell interaction by the four scFvs (2B6, 2D9, 2E7 and 2G3) individually, and as a cocktail. The peptide pre-incubated with the respective antibodies was allowed to bind to the HepG2-hNTCP-C4 cells. A rabbit polyclonal antibody to preS1peptide (21–47 a.a.) and a commercial anti-preS1 mAb were taken as positive controls while an unrelated mAb, 1A7, was taken as a negative control. The first panel shows the DAPI image, second panel shows FITC image and the third panel shows the merged image captured using 60 × objective lens for each treatment (Scale = 10 μm).

The *in vitro* assays to determine the neutralizing potential of the anti-HBs antibodies employ cells that are permissive to HBV infection and express the functional HBV receptor, hNTCP, on their surface (differentiated human or Tupaia hepatocytes or HepaRG cell line). Cell lines such as Huh7 and HepG2 retain hepatocyte specific surface markers and allow HBV binding through preS1 region (21–47a.a.) but are not a good model due to low expression levels of hNTCP. We therefore used a cell line stably transfected with hNTCP (HepG2-hNTCP-C4) for our study. The stable cell line showed higher expression levels of the functional HBV receptor, hNTCP (Fig. 4A,B) and has been shown to be permissive to HBV infection. Using HepG2-hNTCP-C4cells, we have established fluorescence microscopy and flow cytometry based ‘surrogate *in vitro* neutralization assay’ to determine the ‘neutralizing potential’ of preS1 specific antibodies. The ability of these antibodies to block the binding of rec-preS1(60) (Fig. 4C) to the HepG2-hNTCP-C4 cells was taken as a measure of their ‘neutralization potential’. HepG2-hNTCP-C4 cells were incubated with the rec-preS1(60)-FITC conjugate (1 μM). Treatment of the cells with pre-incubated mixture of rec-preS1(60)-FITCand the four scFvs, 2B6, 2D9, 2E7 and 2G3 (10 μM each) individually or as a cocktail (equimolar ratio; 2.5 μM each) showed varying degrees of inhibition of HepG2-hNTCP-C4-preS1(60) interaction (number of FITC stained cells) as evident by the peak shift (from stained population to unstained) in the histogram (Fig. 4D). The number of FITC stained cells was reduced in each case as compared to the cells treated with rec-preS1(60)-FITC alone. Amongst the scFvs, 2E7 showed the best inhibition individually in comparison to 2G3, 2B6 or 2D9. The positive controls, anti-preS1(21–47a.a.) rabbit polyclonal antibody and commercial anti-preS1 mAb raised against preS1 peptide (21–47a.a.) also showed inhibition of the preS1 interaction with cells to a similar extent as shown by the scFv cocktail, while an unrelated mAb, 1A7 showed no inhibition at all. The fluorescence microscopy was carried out on an Olympus Laser Confocal Scanning Microscope (FV1000D) using a 60 × oil objective. The cells were seeded on collagen coated coverslips and treated as done for flow cytometry. The results obtained were in consistence with the flow cytometry data where a high degree of inhibition was achieved using the scFvs as well as cocktail of four antibodies (Fig. 5). The controls also provided consistent results with anti-preS1 mAb and anti-preS1 RpAb showing inhibition of preS1-NTCP interaction while no inhibition was evident with the unrelated mAb 1A7. The results provide a proof of concept for better coverage of a broad epitope using a cocktail of antibodies as against the conventional single monoclonal antibody use.

## Discussion

Neutralizing antibodies have rapidly become a clinically important drug class for prophylaxis of several infectious diseases where they can confer post-exposure protection against the infection^37^,^38^. Blood purified HBIG, alone or in combination with hepatitis B vaccine, is advocated as an adjunct therapy for post-exposure hepatitis B prophylaxis^5^,^7^. However, due to the low specific activity of HBIG and emergence of newer HBV ‘escape’ variants which can persist despite previous immunization, this therapy is frequently rendered ineffective. This underlines the need for better measures for passive prophylaxis^39^,^40^.

Antibodies targeting various epitopes in the immunodominant S region and the pre-S region of the HBV surface antigen have shown promise as neutralizing agents^10^,^41^. Different research groups have mapped the HBV preS1 domain involved in receptor recognition but the HBV preS1 region between residues 21–47 is considered crucial for attachment and uptake of the virions into the hepatocytes^15^,^16^,^18^,^19^. A peptide spanning this pre-S1 region has been used as a hepatocyte targeting agent and demonstrated to inhibit viral binding to human hepatic cells^42^. PreS1 (21–47 a.a.)-specific antibodies have been shown to be effective in prevention of hepatitis B infection in non-human primates (NHPs). Third generation vaccines therefore incorporate a preS1-component to generate a better immune response^10^,^24^,^43^. Antibodies which emerge during the course of acute HBV infection in patients are neutralizing and also initiate HBV clearance. The anti-preS1 responses are taken as a measure of the adequacy of patient’s anti-HBV response and usually precede the responses to S protein but diminish within few months after viral clearance^13^,^44^,^45^. For the assessment of spontaneous recovery from hepatitis B infection in our study, we considered positivity for viral sero-markers - anti-HBc and anti-HBs along with negative HBV-DNA and HBsAg in individuals who had no medical history for anti-HBV therapy^46^. We detected antibodies against S as well as preS1 antigens in these individuals; however, the titers for anti-HBs antibodies were higher in comparison to anti-preS1 antibody titers. Antibodies detected against the preS1-peptide (21–47 a.a.) in the sera were indicative of a protective immune response. The time interval between sample collection and infection was unknown, and the low levels could be due to waning of anti-preS1 responses following recovery^45^. The presence of anti-preS1 antibodies in the serum of these individuals was confirmatory of a recent episode of acute hepatitis B infection followed by recovery and corroborated with previous reports^44^,^47^.

Human monoclonal antibodies have been isolated from PBMCs of healthy individuals or human volunteers immunized with the isolated antigen or from successfully treated/naturally recovered patients using hybridoma/phage display technology^30^,^48^,^49^. Our selected cohort of individuals presents a reasonable probability of obtaining neutralizing antibody. A medium sized phage displayed scFv library was constructed from the RNA of the circulating lymphocytes of these individuals and biopanning and screening of this library yielded four distinct monoclonal antibodies. The four scFvs- 2B6, 2D9, 2E7 and 2G3 had unique sequences but showed similar binding towards the preS1-peptide and the preS1 containing recombinant as well as blood derived antigens. These similar specificities but differing interactions of the four validated mAbs can be attributed to differential germline gene usage and evolution through somatic hypermutations under a rigorous selection pressure for preS1 antigen during affinity maturation process. Affinity maturation involves efficient and productive change of germline Tryptophan, Serine and Tyrosine residues with promiscuous binding residue types in the antigen binding site that confer specificity towards a particular antigen^50^,^51^. The absence of Tryptophan and Tyrosine amongst the peptide interacting residues of the four mAbs is indicative of accumulation of somatic hypermutations in the antibody genes. The differential germline usage amongst the antibodies was reflected in the usage of IGHV6 in 2E7 and 2G3, and IGHV3 and IGHV4 respectively, in 2B6 and 2D9 scFv heavy chains. The diversity in V_L_ germline gene usage was limited to IGKV1 with only 2E7 utilizing IGKV3 gemline light chain gene. Germline analysis also revealed significant genetic variations, especially in the CDRs and FRs, characterized by a high replacement to substitution mutations (R/S) ratio^52^.

Structural or computational studies provide useful insights into the rational design of novel therapeutic strategies^53^. It has been observed that a broadly neutralizing monoclonal antibody identifies its signature epitope and generates a selective immune pressure under which the viruses tend to undergo antigenic drift. This leads to viral mutations at sites that identify the antibody binding site and generation of antibody resistant ‘escape mutants’ which limit the efficacy of monoclonal antibody therapy^54^,^55^. Therefore, a broad and potent polyclonal humoral response against multiple neutralization epitopes is required to control preexisting viral mutants and to limit viral evolution. The *in vivo* immune response against the hepatitis B virus is also polyclonal in nature involving the neutralizing epitopes^13^. Distinct antibodies have differing conformations of the binding site and utilize discrete interaction patterns to recognize a similar epitopic region. This multifaceted viral recognition by antibodies reduces the possibility of viral escape^56^. Docking of the four human mAbs 2B6, 2D9, 2E7 and 2G3 with the preS1-peptide revealed the detailed molecular interactions. Discrete but overlapping set of preS1-peptide residues which span the entire 28-mer peptide were recognized by the four scFvs and provided broad coverage of the defined hepatocyte interaction domain of HBV. The targeted preS1 epitope (21–47 a.a.) is indispensable for the viral infection process and is evolutionarily conserved with minimal variations amongst the viral genotypes. Our findings lend support to the hypothesis that for a broad and better prophylactic intervention against HBV, a panel of antibodies against preS1 region should be used as they would mimic natural immune response providing a broader coverage of viral attachment site.

Although human or tupaia hepatocytes have been frequently used, lack of established cell lines has hampered HBV attachment and replication studies. Host specificity and tissue tropism of HBV are attributed to its specific receptor recognition. Identification of NTCP as a host entry receptor for HBV has vastly enhanced the knowledge of HBV entry and infection process and also paved the way towards development of new host-directed antivirals. The NTCP is significantly expressed in HBV-susceptible cells, PHH and differentiated HepaRG cells but is weakly expressed in hepatic cell lines such as HepG2 and Huh-7 which are refractory to productive HBV infection. It has been demonstrated that overexpression of NTCP in these cell lines can make them susceptible to HBV infection^21^,^22^,^23^. The viral entry process is an attractive target for the development of antiviral agents. Neutralizing antibodies exert antiviral activities by blocking viral entry into cells and/or accelerating viral clearance from circulation^41^. Flow cytometry and fluorescence microscopy based ‘surrogate *in vitro* neutralization assays’ that we used were based on inhibition of peptide attachment to HepG2-hNTCP-C4 cells by the antibodies. The assays provide a rapid, virus-free platform for the assessment of neutralizing efficacy of anti-preS1 antibodies. Our results suggest that while all the antibodies were able to inhibit peptide attachment to the HepG2-hNTCP-C4 cells individually, the combined use of these antibodies could educe an equivalent but broader neutralization with a coverage of a larger epitope region in the surrogate *in vitro* neutralization assays. The antibody panel generated in this study may also have a therapeutic value in established infection. Similar combined usage of antibodies has proven effective as a post-exposure prophylactic for lethal viral diseases with no approved vaccine such as HIV and especially Ebola virus infection in non-human primates^37^,^57^.

Vaccine or drug induced viral escape mutants along with multiple viral subtypes are the major contributors towards the global prevalence of hepatitis B^4^,^40^. Spontaneously recovered individuals have natural neutralizing antibodies in their blood and we present a method to efficiently harness these natural human antibodies. Though we have not explored the idea *in vivo*, the marked difference in the antibody sequences resulting in very distinct epitope-paratope interactions raises the possibility that use of a cocktail of antibodies would be more tolerant to genotypic variations/mutations in the preS1 region. We propose that while mimicking the natural polyclonal response, such panel of antibodies would provide us with an efficient and well characterized way of viral neutralization.

## Methods and Materials

### Reagents

The vectors pAK100 and pAK400 were received as a kind gift from Dr. Andreas Plückthun, Department of Biochemistry, University of Zurich, Zurich, Switzerland. Yashraj Biotech, India provided the purified blood-derived native HBsAg as a gift. *E. coli* strains TG1 and HB2151 were obtained from Amersham Biosciences.

### Cell culture

HepG2-hNTCP-C4 cells were cultured in collagen coated flasks (0.1 mg/mL collagen [Sigma, C3867]) as described earlier at 37 °C in a humidified incubator containing 5% CO_2_. The growth media consisted of HEPES buffered DMEM (Invitrogen, 2320032) supplemented with 200 units/ml penicillin, 200 μg/ml streptomycin (Gibco, 15140-122), 10% FBS (Gibco, 16000-044), 50 μM hydrocortisone (Himedia, TC344), 5 μg/ml insulin (Himedia, TC190) and 400 μg/ml G418 (InvivoGen, ant-gn-1).

### Construction of human scFv library

#### Selection criteria and serum ELISA

All ethical approval for the study was obtained from the institutional (AIIMS, New Delhi) ethics committee (IESC/OT-02/02.07.10). All methods were carried out in accordance with the approved guidelines of the AIIMS ethics committee. Also it is confirmed that an informed consent was obtained from all the subjects before enrolment into the study.

Individuals who had naturally recovered from hepatitis B but were on routine check-up in the Liver clinic of All India Institute of Medical Sciences (AIIMS) were recruited. The selection criteria included chance detection cases with positivity for anti-HBc and anti-HBs antibodies but with no detectable amounts of HBsAg or HBV-DNA and no history of therapy for infection. 10 ml blood collected from all the individuals (n = 7), was used for serum separation as well as peripheral blood lymphocyte (PBL) isolation (using Histopaque-1077). For serum ELISA, serum (1:100) was incubated with the antigens immobilized on Nunc maxisorp plates (Nunc, 442404) at 37 °C for 1 hour and the bound IgG antibodies were detected using anti-human IgG-HRP conjugate (1:5000) and substrate OPD.

#### Phage displayed single chain variable fragment (scFv) library construction

RNA isolated from PBLs was reverse transcribed to cDNA and the entire variable region repertoires of human heavy and light chains were amplified using consensus forward and reverse primers using multiple PCR reactions for each chain (24 reactions for V_H_, 21 for V_L_λ and 30 for V_L_κ). The heavy and light chain primers (IDT) were modified from primers reported by Pansri *et al.*, 2009, wherein the NotI restriction site was replaced by SfiI restriction site. Separate pools of V_H_, V_L_λ and V_L_κ repertoires were made by pooling amplified variable region products (~400 bp). The scFv (~800 bp) was constructed by overlapping the V_H_ and V_L_ pools with a flexible linker (Gly_4_Ser)_3_ using ‘Assembly PCR’ and Final ‘Pullthrough’ PCR. The final library was constructed by ligating the scFv into pAK100 phagemid vector, transformation into TG1 competent cells and pooling together the resulting colonies^30^,^58^,^59^.

### Biopanning

The phage rescue from the constructed library was carried out as described earlier using VCSM13 helper phage (Stratagene, 200251)^58^. Three rounds of biopanning were carried out using biotinylated preS1-peptide (5 μg/ml) immobilized on high binding streptavidin coated plates (Pierce, 15501). Library phages (~1 × 10^12^) were incubated with the peptide and unbound phages were washed out with PBST (0.1%). The bound phages were eluted using 100 μl of 200 mM glycine-HCl, pH 2.2 and neutralized using 15 μl 1 M Tris-HCl, pH 9.1. The eluted phages were infected into mid-exponential phase (OD_600_ ~ 0.4) TG1 cells and grown for 1 hour at 37 °C and plated on 2 × YT/ Chloramphenicol/2%Glc/ Agar plates and incubated overnight at 37 °C. Washing stringency was gradually increased over each round (Round 1–0.1% Tween-PBS × 10 washes; Round 2–0.3% Tween-PBS × 15 washes; Round 3–0.5% Tween-PBS × 20 washes) to wash out the low affinity phages. The number of input and output phages for each round was calculated by making a transformation unit count. The colonies obtained after the third round were individually grown as monoclonals.

### Screening and characterization of scFv clones

For screening, 100 μl of supernatant containing rescued phage from each monoclonal (scFv) was added to the blocked streptavidin-coated plate coated with biotinylated preS1-peptide (5 μg/ml) and incubated for 1 hour at 37 ^o^C. Post washing, the bound phage was detected using 100 μl of anti-M13 HRP-conjugated antibody (GE lifesciences, 27942101) (1:2000) using TMB as substrate. For further characterization, the phage containing supernatant was precipitated using PEG/NaCl and used in ELISA in a concentration of 1 × 10^12^ phages/100 μl.

The scFv gene of selected scFvs was cloned into pAK400 vector and transformed into HB2151 *E. coli* cells for periplasmic expression. The scFv clone was then grown in 1L 2xYT/Chloramphenicol media and induced at an OD~0.8 with 1 mM IPTG (Amresco, 0487) for 15 hours at 20 °C, 250 rpm. The periplasmic extract was prepared using ‘Osmotic Shock method’^60^. 100 μL of supernatant was added to the preS1-peptide coated streptavidin plate and incubated for 1 hour at 37 °C. Post washing, bound phages were detected using 100 μl of anti-his antibody (1:2000) (Cell Signaling Technologies, 3724) and anti-rabbit HRP-conjugated antibody (Cell Signalling Technologies, 70745) using TMB as substrate.

### Protein Purification and labeling

Recombinant preS1 antigens: preS1(60) and preS1(108) were cloned in pET32a + vector between NcoI and XhoI restriction sites and the expression constructs were transformed into BL21(DE3) *E. coli* cells. The antigens were expressed in 2×YT culture medium containing ampicillin and induced at O.D. ~ 0.8 with 1 mM IPTG at 20 °C for 15 hours. The recombinant proteins were purified from the soluble (cytoplasmic) fraction in a single purification step by Ni-NTA affinity chromatography. FITC labeling of the purified preS1(60) was done using a commercial kit (Thermo Scientific, 53027) according to manufacturer’s instructions. For scFv purification, periplasmic fraction of HB2151 *E. coli* cells transformed with scFv-pAK400 expression construct was utilized. The scFvs were purified using his-tag by Ni-NTA affinity chromatography.

### Molecular modeling of antibody fragments (scFvs) and preS1-peptide

The sequencing of the scFvs was done by Invitrogen using pullthrough primers. The antibodies were numbered as per Kabat rule. The germline sequences of V_L_ and V_H_ were analyzed by online V-Quest software provided by the international ImMunoGeneTics database (IMGT)^31^,^32^ (http://www.imgt.org/IMGT_vquest/share/textes/). The localization of Complementary Determining Regions (CDRs) and Framework regions (FRs) were analyzed for their percent variation and mutation from the original germline sequence.

Molecular models of the scFvs were generated by web based antibody modeling software WAM^33^ (http://antibody.bath.ac.uk/) using ‘dead end elimination’ algorithm for side chain building and Valence Force Field (VFF) screen for final screening. In cases where WAM model could not be predicted, the Prediction of ImmunoGlobulin Structure (PIGS) server was used for building the scFv model using default parameters^34^ (http://arianna.bio.uniroma1.it/pigs). The molecular model of preS1-peptide was predicted using I-TASSER of threading method^35^ (http://zhanglab.ccmb.med.umich.edu/I-TASSER/). The server generated five models and the best one was selected based on C-score. All the predicted models were viewed and analyzed in Pymol viewer.

### Molecular Docking of the pre-S1 peptide and scFvs

Web based server ‘*PatchDock*’ was used for the peptide-scFv docking studies^36^ (http://bioinfo3d.cs.tau.ac.il/PatchDock/). Molecular models of scFv and preS1-peptide were used as input in the antigen-antibody docking algorithm. Twenty models were generated by the server and the top ten solutions with a near-native conformation were identified. The server ranked the models on the basis of geometric score, desolvation energy, interface area size and the actual rigid transformation of the solution. The highest ranked model was selected for analysis.

### Cellular binding inhibition assays

HepG2-hNTCP-C4 cells (~5 × 10^4^ cells) were grown on 12-mm coverslips coated with 0.1 mg/mL collagen (Sigma) for 24 hours, washed with 1 × PBS and incubated with either 1 μM preS1(60)-FITC protein or with preS1(60)-FITC -scFv mixtures at room temperature for 30 min. The preS1(60)-FITC protein (1 μM) and the scFv (10 μM for each scFv or control antibodies) were pre-equilibrated for 1 hour at room temperature in DMEM containing 0.5% FBS. (For Cocktail, each scFv was taken at a concentration of 2.5 μM to get a final cocktail concentration of 10 μM). The cells were washed with PBS three times, fixed with 4% (w/v) paraformaldehyde (PFA) and the coverslip was mounted using Fluoroshield^™^ mounting media with DAPI (Sigma, F6057) on a glass slide. The fluorescence microscopy was performed on an Olympus Laser Confocal Scanning Microscope (FV1000D) using a 60 × oil objective.

For flow cytometry experiments, 5 × 10^4^ cells were seeded in collagen coated 24-well plate (Corning, 354408), grown at 37 °C for 24 h, treated and fixed similar to confocal microscopy. The samples were acquired using FACS Verse flow cytometer (BD Biosciences) and the analysis was performed on FlowJo software.

## Additional Information

**How to cite this article**: Sankhyan, A. *et al.* Inhibition of preS1-hepatocyte interaction by an array of recombinant human antibodies from naturally recovered individuals. *Sci. Rep.* **6**, 21240; doi: 10.1038/srep21240 (2016).

## Figures and Tables

**Table 1:** Analysis of HuMAbs selected against preS1-peptide region (21–47a.a.) for their germline origin.

HuMAb clone	Germline genes				
	VH			VL	
	V	D	J	V	J
2B6	IGHV3-11^*^01	IGHD2-2^*^01	IGHJ1^*^01	IGKV1-12^*^01	IGKJ3^*^01
2D9	IGHV4-b^*^02	IGHD7-27^*^01	IGHJ5^*^02	IGKV1-27^*^01	IGKJ1^*^01
2E7	IGHV6-1^*^01	IGHD1-14^*^01	IGHJ6^*^02	IGKV3-20^*^01	IGKJ2^*^01
2G3	IGHV6-1^*^01	IGHD3-10^*^02	IGHJ4^*^02	IGKV1-12^*^01	IGKJ4^*^01

**Table 2:** Interacting residues of the preS1-peptide and heavy and light chains of the four scFvs.

2G3			2B6			2D9			2E7		
Peptide residue	scFv residue	Location	Peptide residue	scFv residue	Location	Peptide residue	scFv-residue	Location	Peptide residue	scFv residue	Location
Ser 18	H99Asp	CDRH3	Asn17	L1Glu, L2Thr	FRL1	Asn17	H56Ser, H57Thr	CDRH2, CDRH2	Asn19	L27ASer	CDRL1
Gly3	H50Arg	CDRH2	Asn20	H61Asp, H64Lys	FRH3	Asn20	H34Tyr	CDRH1	Ser18	L29Ser	CDRL1
Gly3	H52Tyr	CDRH2	Asp22	H64Lys	FRH3	Asp22	L91Tyr, L93Ser	CDRL3, CDRL3	Pro27	H57Asn	CDRH2
Gly3	L91Val	CDRL3	Asn26	H52Ser	CDRH2	Pro27	L28Gly	CDRL1			
Phe4	L94Phe	CDRL3	Pro27	H52Ser	CDRH2						
Asp11	H53Ser	CDRH2	Ala16	L92Asp	CDRL3						
Asp7	H56Tyr	FRL3	Ser18	L95Pro	CDRL3						
